# Saxitoxin Modulates Immunological Parameters and Gene Transcription in *Mytilus chilensis* Hemocytes

**DOI:** 10.3390/ijms160715235

**Published:** 2015-07-06

**Authors:** Allisson Astuya, Crisleri Carrera, Viviana Ulloa, Ambbar Aballay, Gustavo Núñez-Acuña, Hélène Hégaret, Cristian Gallardo-Escárate

**Affiliations:** 1 Laboratory of Cell Culture and Marine Genomics, Marine Biotechnology Unit, Faculty of Natural and Oceanographic Sciences and Program COPAS Sur-Austral, University of Concepción, Concepción 4070386, Chile; E-Mails: aastuya@udec.cl (A.As.); crislericarrera@udec.cl (C.C.); viulloa@udec.cl (V.U.); amaballay@udec.cl (A.Ab.); 2 Laboratory of Biotechnology and Aquatic Genomics, Interdisciplinary Center for Aquaculture Research (INCAR), University of Concepción, Concepción 4070386, Chile; E-Mail: gustavonunez@udec.cl; 3 Laboratoire des Sciences de l’Environnement Marin (LEMAR), UMR 6539 CNRS/UBO/IRD/IFREMER, Institut Universitaire Européen de la Mer, Technopole Brest Iroise, 29280 Plouzané, France; E-Mail: helene.hegaret@univ-brest.fr

**Keywords:** saxitoxin, hemocytes, immune response, paralytic shellfish poisoning, reactive oxygen species (ROS)

## Abstract

Saxitoxin (STX) is a neurotoxin produced by dinoflagellates in diverse species, such as *Alexandrium* spp., and it causes paralytic shellfish poisoning (PSP) in humans after the ingestion of contaminated shellfish. Recent studies have suggested that the immune functions of bivalves could be affected by harmful algae and/or by their toxins. Herein, hemocytes are the main effector cells of the immune cellular response. In this study, we evaluated the response of hemocytes from the mussel *Mytilus chilensis* to STX exposure in a primary culture. Cell cultures were characterized according to size and complexity, while reactive oxygen species (ROS) production was evaluated using a dichlorofluorescein diacetate (DCFH-DA) assay. Finally, phagocytic activity was measured using both flow cytometry and fluorescence microscopy assays. Additionally, gene transcription of candidate genes was evaluated by qPCR assays. The results evidenced that exposures to different concentrations of STX (1–100 nM) for 24 h did not affect cell viability, as determined by an MTT assay. However, when hemocytes were exposed for 4 or 16 h to STX (1–100 nM), there was a modulation of phagocytic activity and ROS production. Moreover, hemocytes exposed to 100 nM of STX for 4 or 16 h showed a significant increase in transcript levels of genes encoding for antioxidant enzymes (*SOD*, *CAT*), mitochondrial enzymes (*COI*, *COIII*, *CYTB*, *ATP6*, *ND1*) and ion channels (*K^+^*, *Ca^2+^*). Meanwhile, *C-type lectin* and *toll-like receptor* genes revealed a bi-phase transcriptional response after 16 and 24–48 h of exposure to STX. These results suggest that STX can negatively affect the immunocompetence of *M. chilensis* hemocytes, which were capable of responding to STX exposure *in vitro* by increasing the mRNA levels of antioxidant enzymes.

## 1. Introduction

Harmful algal blooms (HABs) are excessive accumulations of phytoplankton that produce biotoxins, which can adversely affect humans, animals and ecosystems. The effects of HABs on the physiology of bivalves can result for example in reductions in filtration activity, changes in valve closure, paralysis of the adductor muscle, mantle retraction, increases in mucus production or erratic cardiac activity [1,2,3,4,5,6]. There are differences in the resistance of different species of bivalves to STX [7], which also is associated with differences in the ability to accumulate these toxins [8,9]. Although *Mytilus* spp. are considered refractory to contamination with paralytic shellfish toxins (PST), accumulating high concentrations of toxins in their tissues, there are reports of negative effects of PST in these bivalves [10,11,12]. In addition to this, histopathological lesions occur in bivalves exposed to HAB species through the degranulation and diapedesis of hemocytes into the alimentary canal and through hemocyte migration into connective tissue, which suggests an immune response similar to the inflammation process [2,13,14].

In bivalves, the humoral and cellular immune responses are mediated by hemocytes and involve diverse cellular processes, such as phagocytosis, reactive oxygen species (ROS) production, opsonization, nodule formation and the release of immune mediators [15]. *In vivo* and *in vitro* studies have reported changes in cell morphology, increases in mortality, reductions in phagocytosis, changes in adhesion and ROS production in hemocytes after exposure to marine toxins derived from HABs, thereby indicating that the immune response to toxic microalgae could be directly related to toxin levels [16,17,18]. In addition to cellular immune parameters, changes occur at the transcriptional level of stress response genes in hemocytes exposed to marine toxins [16,17,19]. Furthermore, a transcriptional response was observed in *Mytilus chilensis* exposed *in vivo* to saxitoxin (STX), specifically through an increase in the mRNA levels of *superoxide dismutase* (*SOD*), *catalase* (*CAT*), *ferritin* and *heat-shock protein* genes, while, to a lesser extent, *ependymin*, *fibrinogen-like*, *galectin* and *mytilin B* genes evidenced differentiated expressions after toxin exposure [20]. A complete review summarizing the molecular responses of bivalve species to phycotoxins was published in 2012, including the effects of STX exposure in marine environments [21]. Nonetheless, previous studies are focused only on *in vivo* assays and do not include *in vitro* assays, which would be helpful in developing standardized methods for evaluating the responses of diverse organisms to marine toxins and HABs. Furthermore, the fact that this species is considered a sentinel species for biomonitoring and even with the high amount of knowledge regarding the “omics” approaches to marine bivalves, there are more expectations for integrative studies focusing the response of marine bivalves on xenobiotics [22]. In this context, the integration of *in vivo* and *in vitro* approaches is still scarce.

In Chile, *Alexandrium* spp*.* are the species most associated with HABs [23,24,25]. Species of the genus *Alexandrium* are the main producers of paralytic shellfish poisoning (PSP) toxins, with saxitoxin as the most toxic analogue [26,27]. Studies using *M. chilensis* exposed to *Alexandrium catenella* have shown that this species is capable of a rapid response and has a great capacity for toxin acclimation. The mussel *M. chilensis* is of particular interest given its ecological and commercial importance to the Austral Region of Chile, where over 180,000 tons are annually produced through suspended cultivation and where HABs are very frequent [28]. Therefore, *M. chilensis* could be a good model to study the effects of STX on hemocytes.

The aim of this study was to evaluate the effects of saxitoxin on cellular and molecular responses in a primary culture of hemocytes from *M. chilensis*. For this, we analyzed cellular parameters, such as phagocytosis and ROS production, and changes at the transcriptional level for antioxidant enzymes, mitochondrial proteins, ion channels and pattern recognition receptor (PRR).

## 2. Results

### 2.1. Primary Culture of Hemocytes from M. chilensis

**Figure 1:**
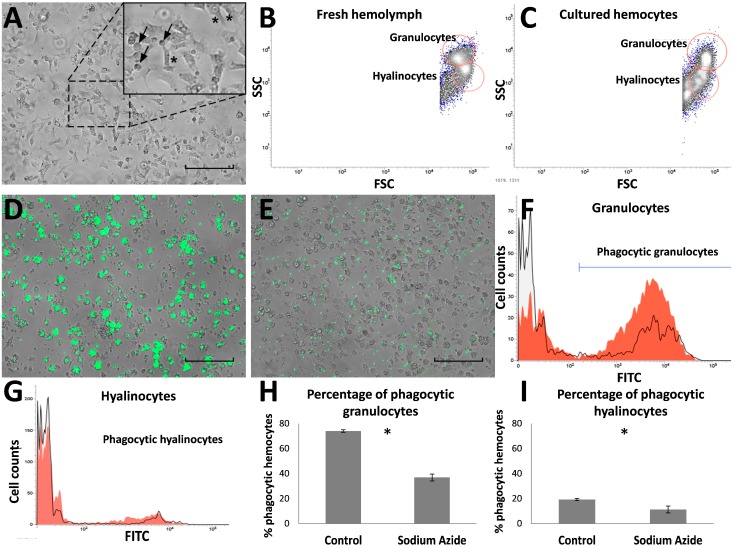
Characterization of *Mytilus chilensis* hemocytes in primary cell culture. (**A**) Light microscopy image. Insert: Granulocyte-type hemocytes (asterisks) and hyalinocyte-type hemocytes (arrows), Scale bar = 100 μm; (**B**,**C**) Flow cytometry analyses of fresh (**B**) and cultured (**C**) hemocytes showing both granulocyte-type and hyalinocyte-type hemocytes appearing in the red circles; (**D**) Fluorescence microscopy image of phagocytosis assay on hemocytes incubated with 0.2% FITC-labeled zymosan (green) for 1 h, Scale bar = 100 μm; (**E**) Inhibition of phagocytosis with 2% sodium azide, Scale bar = 100 μm; (**F**,**G**) Phagocytosis of 0.2% FITC-labeled zymosan after 1 h observed in granulocytes (**F**) and hyalinocytes (**G**). The red lines correspond to phagocytosis of control hemocytes, and the overlaid black lines correspond to phagocytosis of hemocytes incubated with sodium azide showing inhibition; (**H**,**I**) Effect of 2% sodium azide on the phagocytic activity of granulocyte-type (**H**) and hyalinocyte-type (**I**) hemocytes. Bars represent the mean ± standard errors (*n* = 4). * *p* < 0.05.

Hemocytes cultured from *M. chilensis* had a round-shape or a flattened polygonal shape with granules (Figure 1A), and these adhered to the substrate 3 h after seeding. According to the morphological appearance of the cultured hemocytes, two main groups were identified, which could correspond to hyalinocyte and granulocyte-type cells. Hemocytes in the culture were viable up to seven days, as assessed by an MTT assay (data not shown). Hemocyte visualization by size (forward scatter, FSC) and internal complexity (side scatter, SSC) showed a distribution similar to a fresh hemolymph of *M.* *chilensis* (Figure 1B). Two separate hemocyte populations were observed in the primary cell cultures (Figure 1C). Granulocyte-type cells corresponded to 39.9% ± 0.6% of total events analyzed. Hyalinocyte-type cells appeared less granular, smaller in size and represented 60.1% ± 0.4% of the total events.

On the other hand, analysis using fluorescence microscopy, as assayed using FITC-labeled zymosan particles, showed an evidently higher percentage of phagocytic hemocytes in controls than in hemocytes co-incubated with 2% sodium azide, a phagocytosis inhibitor (Figure 1D,E). Flow cytometry analysis evidenced different phagocytic activity for granulocytes and hyalinocytes after incubation with zymosan-FITC particles and co-incubation with zymosan-FITC and sodium azide (Figure 1F,G). Phagocytic cells represented 74.0% ± 0.9% of the granulocytes, and phagocytic activity decreased to 36.9% ± 2.7% with sodium azide. In contrast, phagocytic hyalinocytes accounted for 19.3% ± 0.5%, and phagocytic activity decreased to 11.3, while phagocytic activity decreased to 0.7% with sodium azide.

### 2.2. Effects of Saxitoxin (STX) on Cellular Parameters

**Figure 2:**
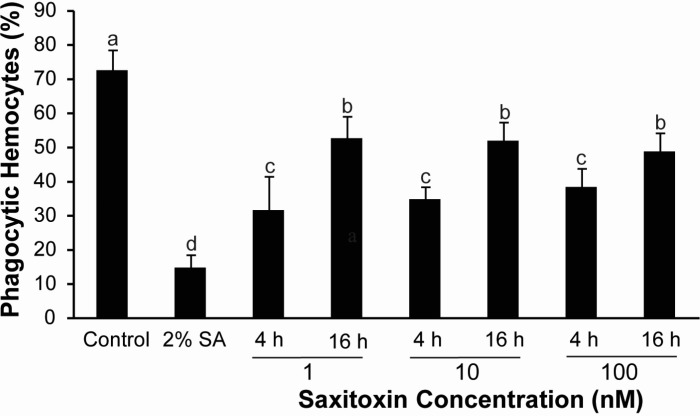
Effect of saxitoxin (STX) on the phagocytic activity of hemocytes. Hemocytes were pre-incubated with STX for 4 or 16 h and then incubated with 0.2% FITC-labeled zymosan for 1 h at 20 °C or co-incubated with 2% sodium azide. Phagocytic cells were analyzed by fluorescence microscopy. Bars represent the mean ± standard errors (*n* = 3 cultures of hemocytes). Different superscripts indicate statistically-significant differences (*p* < 0.05; Kruskal–Wallis/Mann–Whitney) at 4 and 16 h after incubation with STX as compared to the control group. SA: Sodium azide.

The percentage of phagocytic cells in the control condition was 72.3%, whereas phagocytosis was inhibited at 80% with sodium azide. A decrease in the percentage of phagocytic cells was observed after 4 and 16 h of exposure to STX (Figure 2). The percentage of phagocytic cells was reduced by approximately 30% after 4 h and by 50% after 16 h of incubation with STX, compared to the control. However, there was no dose-dependent effect of STX (Figure 2).

**Figure 3:**
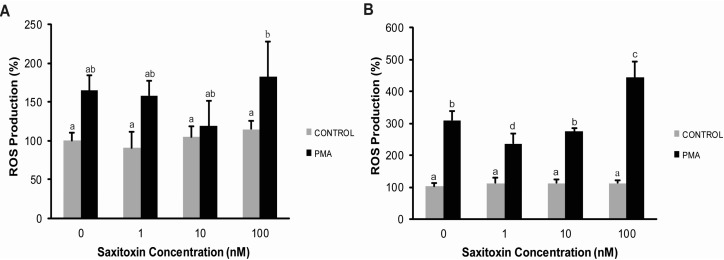
Evaluation of STX effects on reactive oxygen species (ROS) production in hemocytes. Cells were incubated with STX for (**A**) 4 h or (**B**) 16 h and later incubated with 10 µM dichlorofluorescein diacetate (DCFH-DA) for 90 min. PMA: Cells stimulated with 10 µg·mL^−1^ PMA. Bars represent the mean ± standard errors (*n* = 3 cultures of hemocytes). Different superscripts indicate statistically significant differences (*p* < 0.05) at 4 (ANOVA/Tukey HSD) and 16 h (Kruskal–Wallis/Mann–Whitney) after incubation.

On the other hand, hemocytes stimulated with phorbol myristate acetate (PMA) showed a higher production of intracellular ROS at 4 and 16 h, compared to hemocytes incubated without PMA. At 4 h, there was no significant effect of STX in PMA-stimulated hemocytes (Figure 3A). However, a decrease in ROS production was observed at 16 h in cells exposed to 1 and 10 nM STX, but there was an increase at 100 nM STX (Figure 3B).

### 2.3. Effects of STX on the Gene Expression of Antioxidant Enzymes, Oxidative Phosphorylation Transcripts, Ion Channels and Pattern Recognition Receptors

**Figure 4:**
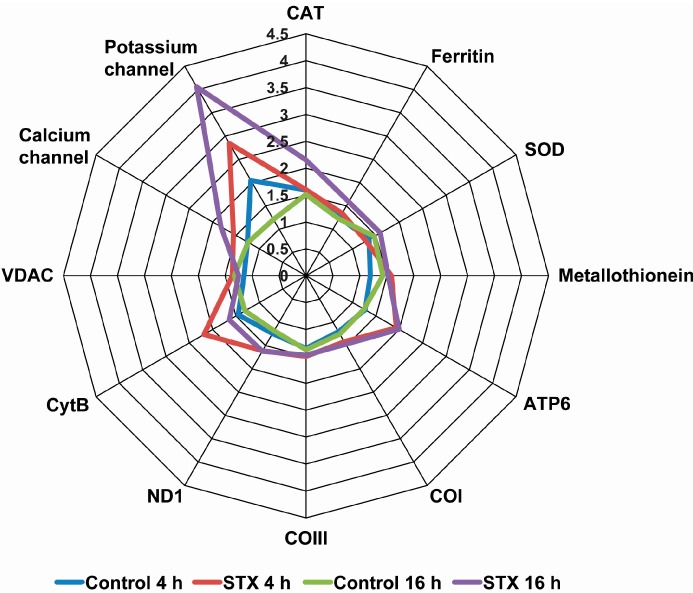
Radial graph of relative transcript levels for studied genes in cultured hemocytes after exposure to STX or a control solution. Cells were seeded in six-well plates, and after three days of culturing, cells were incubated with 100 nM STX for 4 or 16 h.

Gene transcription levels of antioxidant enzymes showed a significant increase after the incubation of hemocytes with 100 nM STX for 16 h. The mRNA levels of *SOD*, *CAT* and *ferritin* increased after 16 h, while the expression level of *metallothionein* increased at 4 and 16 h. Besides this, hemocytes incubated with STX, regardless of concentration or time, showed similar expression levels of mitochondrial enzymes, such as *COI*, *COIII*, *CytB*, *ATP6* and *ND1*, which were more expressed than in cells without STX at both times analyzed. In the case of ion channel genes, increased expression levels were observed at 4 and 16 h for *K^+^* and *Ca^2+^ channels*. This was especially evidenced for the *K^+^ channel* at 16 h, which showed a four-fold increase as compared to control cells. Conversely, *VDAC* transcripts evidenced decreased expression levels after 16 h of exposure to STX (Figure 4). Nucleotide alignments and BLAST analyses evidenced that these last three transcripts corresponded to the subtype of “voltage-dependent channels”, also known as “voltage-gated channels”.

**Figure 5:**
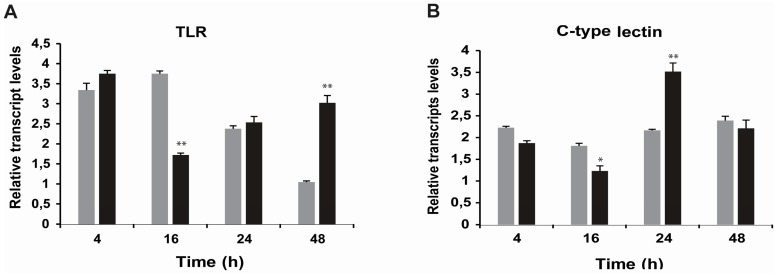
Relative transcript levels for studied genes (TLR (**A**) and C-type lectin (**B**)) of the pattern recognition receptor (PRR) in cultured hemocytes after exposure to STX. Cells were seeded in six-well plates, and after three days of culturing, cells were incubated with 100 nM STX for 4, 16, 24 or 48 h. Black bars = Challenged hemocytes (STX exposed); grey bars = Control hemocytes (mean ± SE, *n* = 4 cultures of hemocytes). * *p* < 0.05 and ** *p* < 0.01 represent statistical differences at 4, 16, 24 and 48 h after incubation with STX as compared to the control group (ANOVA/Tukey HSD).

With respect to *C-type lectin* and *toll-like receptor* genes, the transcriptional activity revealed a bi-phase transcriptional response that decreased after 16 h, but then increased again from 24–48 h post-exposure to STX (Figure 5).

The transcript annotated as *TLR* that was evaluated by qPCR in this study corresponded to an orthologous sequence to the *TLR-b* gene reported by Toubiana *et al.* [29]. This transcript shared 99.8% of identity with the gene described for *Mytilus galloprovincialis* (GenBank Accession Number JX173687)*.* On the other hand, the *C-type lectin* gene corresponded to the subtype *GalNAc/Gal-specific lectin*.

## 3. Discussion

### 3.1. Characterization of Hemocyte Primary Cell Cultures

Cultured hemocytes mainly showed two morphological types: (i) Larger cells with star and spindle shapes with the clear presence of granules and signs of adherence to the culture surface; and (ii) Less adherent spherical cells. These cell types would correspond to granulocyte-like and hyalinocyte-like cells described from other primary cultures of molluscan hemocytes [30,31]. Flow cytometry analysis strongly supported this hypothesis, by evidencing that the cell types found in this study were similar to those observed in samples from a fresh *M.* *chilensis* hemolymph and other previously reported mussel hemocytes [15,32,33,34,35].

Although morphological microscopy analysis showed these primary cultures to be enriched with granulocyte-like cells, flow cytometry quantification provided a lower proportion of this cell type (data not shown). These results are similar to previous studies showing a 40% decrease in the total number of hemocytes when analyzed by flow cytometry [36]. Although flow cytometry analysis showed a lower proportion of granulocyte-like cells, these cells exhibited higher phagocytic activity, supporting the assumption that these differences could be directly related to the processing of samples for flow cytometry, which could produce the loss of granulocyte-like cells. Furthermore, another validation for the functionality of our primary cultures was conducted to measure the activation of the immune response to the PMA inductor [37,38,39], which indicated a significant increase in ROS production after stimulation.

### 3.2. Cellular Response of Hemocytes to STX Exposure

Regarding the effect of STX on the primary culture of hemocytes, an absence of the cytotoxic effects can be noted between the dosage range of 1–100 nM over 24 h of exposure. These results are consistent with results from *in vitro* studies on fresh hemocytes, although these studies were performed with lower toxin concentrations and shorter incubation times [16,17].

STX did however affect hemocyte functions. The inhibitory effect of STX on nearly 50% of active phagocytic cells at 4 h, as determined by fluorescence microscopy, was comparatively higher than the effects observed in another study using isolated hemocytes and flow cytometry [16], thus supporting the assumption that there was a loss of activated cells during sample handling for flow cytometry assays [36]. Basal ROS production of unstimulated cells incubated with STX was not affected under any of the evaluated conditions, whereas a significant inhibition of ROS production was previously observed in unstimulated *C. gigas* hemocytes after STX incubation [16]. Taking into account the absence of STX effects on the basal ROS production of hemocytes, the stimulation of hemocytes with PMA was performed in order to analyze the STX effects. The results of this evaluation showed changes on ROS production in PMA-stimulated cells incubated with STX at 16 h.

### 3.3. Effects of STX on Gene Transcription in M. chilensis Hemocytes

Undoubtedly, the most noticeable result of transcriptional analyses was the contrasting trend observed for the different voltage-gated ion channels. On the one hand, a decrease in the transcription levels of *VDAC* was observed at 16 h post exposure. The *VDAC* channel is one of the most important modulators of mitochondrial metabolism at the cellular level [40]. This would explain the absence of significant differences in the transcription levels of other mitochondrial genes, such as *ATP6*, *COI*, *COIII* and *ND1*, as well as the decrease in the transcription levels of *CytB* after 16 h of toxin exposure. On the other hand, transcription of calcium and potassium channels increased after 4 and 16 h of exposure to STX. This could be explained by the sodium channel not being the only channel to respond to STX, where instead, there is also a response of other channels. Moreover, the interactions of nucleotide mutations localized in sequences that encode ion channels [41] have also been demonstrated. Another hypothesis could be that this increased transcription of calcium and potassium channels is indeed an attempt to maintain cellular ion homeostasis.

With regards to the other measured genes, an overexpression of antioxidant transcripts (*SOD*, *CAT* and *ferritin*) was observed after STX exposure. This is consistent with previous research showing a similar gene pattern in mussels exposed *in vivo* to STX [20]. Furthermore, pattern recognition receptors (PRR), such as *C-type lectin* and *toll-like receptor*, revealed a bi-phase transcriptional response, decreasing after 16 h and then increasing after 24–48 h exposure to STX. Although the study of PRR against marine toxins is limited, the recognition process through these pathogens and PAMP receptors in marine invertebrate hemocytes has already been described [42,43,44,45]*.* Overexpression of genes might suggest that STX is recognized as an invasive foreign molecule by the immune system of *M. chilensis*, which could act as a PAMP-type molecule. Nonetheless, further analyses are needed to establish a quantitative relationship between *in vitro* and *in vivo* models given the different experimental approaches, and, in this case, the different geographic zones of sampled species.

### 3.4. Use of Primary Hemocyte Cultures in Toxicological Studies

Establishing a primary culture of bivalve hemocytes allows for the analysis and long-term monitoring of different environmental pollutants and inductors under controlled conditions, thereby increasing accuracy and reproducibility [29,46,47,48,49,50]. Several authors have shown that stimulation of fresh hemocytes for 4 h is sufficient to evaluate the response to different environmental stressors, such as toxins, metals and chemical pollutants [47,51,52,53]. However, the use of fresh hemocytes limits the investigation to only a few hours. This long-term *in vitro* model thus also allows a more sensitive assay. Indeed, results after 4 h of *in vitro* incubation showed no significant effects of STX, which indeed appeared to demonstrate the regulation of cell function in *M. chilensis* hemocytes after 16 h of STX exposure. Thus, in addition to accuracy and reproducibility, the establishment of the long-term primary culture of bivalve hemocytes also confers a more sensitive assay to demonstrate the impact of any environmental pollutants and inductors, via regulation of hemocyte function after several hours’ exposure. Future usage of cell cultures could also clarify the mechanisms used by *M. chilensis* to bioaccumulate high concentrations of toxins, unlike other bivalves, such as oysters [28,54]. Additionally, by using primary cell cultures of hemocytes, early biomarkers of pollution could be identified and subsequently used in sentinel organisms, such as *M. chilensis*.

## 4. Experimental Section

### 4.1. Primary Culture of Hemocytes

The primary culture protocol was modified from Cao *et al.* [30,55]. Mussels *M. chilensis* averaging 8 cm in shell length were used; and these were kept on ice during hemolymph collection. Hemolymph was extracted from the adductor muscle using a 25Gauge needle and a syringe and was placed into centrifuge tubes with Alseve’s buffer (382 mM NaCl, 27 mM sodium citrate, 11.5 mM EDTA and 115 mM glucose, pH 7). Then, the hemolymph was centrifuged at 600× *g* for 10 min at 4 °C. The supernatant was removed, and the pellet was resuspended in an L-15 culture medium supplemented with 350 mM NaCl, 7.2 mM KCl, 5.5 mM CaCl_2_, 8.3 mM MgSO_4_, 40 mM MgCl_2_, 115 mM glucose, 2 mM l-glutamine, 300 IU·mL^−1^ penicillin, 300 µg·mL^−1^ streptomycin, 30 µg·mL^−1^ gentamicin and 0.45 mg·mL^−1^ amphotericin-B, pH 7.4. A total of 60 × 10^6^ cells were obtained from approximately 20 mussels. Hemocytes were plated in a cellular density range of 2–4 × 10^6^ cells·mL^−1^ and kept at 4 °C. The experiments were performed on the third day of culture.

### 4.2. Experimental Design

For different analyses, hemocytes were incubated for 4 or 16 h with STX at final concentrations of 1, 10 or 100 nM. The solution of STX dihydrochloride (NRC-Canada, Ottawa, ON, Canada) was prepared by diluting a standard solution in supplemented L-15. Cells were incubated at 4 °C in the dark.

### 4.3. Viability and Phagocytosis Assays Using Light and Fluorescent Microscopy

To determine hemocyte viability, 6 × 10^5^ hemocytes were seeded into 96-well plates and maintained in culture at 4 °C. After 3 days of culture, hemocytes were incubated for 24 h with a solution of 2.5 mg/mL 3-(4.5-dimethylthiazol-2-yl)-2.5-diphenyltetrazolium bromide (MTT; Life Technologies, Eugene, OR, USA). The medium was removed, and 100 µL of dimethyl sulfoxide were then added into each well. The plates were read using a microplate reader (Synergy H1, BioTek, Winooski, VT, USA) at 570 nm.

For phagocytosis assays, three replicates of 1 × 10^6^ cells were plated into 24-well plates. FITC-labeled particles of zymosan (Life Technologies) were added to a final concentration of 0.2%. Sodium azide (2%), a phagocytosis inhibitor [56], was used as a negative control. Plates were maintained in the dark at 4 °C for 1 h. Finally, three images per replicate were taken on a fluorescence microscope (Floid^®^ Cell Image Station, Life Technologies). To assess the percentage of phagocytosis, 200 cells per well were counted.

### 4.4. Hemocyte Phagocytosis Using Flow Cytometry

Flow cytometry analyses were performed in order to assess the phagocytosis rate. Thus, four replicates of 1 × 10^6^ hemocytes from each well were harvested using trypsin (0.25%) after incubation with zymosan particles. Cells were resuspended in supplemented L-15, transferred to 5 mL flow-cytometer tubes and kept on ice. Flow cytometry analyses were performed using a FACSAria III flow cytometer (BD Biosciences, San José, CA, USA), with 10,000 events analyzed per sample. Data were later analyzed using the flow cytometry analysis software BD FACSDiva (BD Biosciences).

### 4.5. Intracellular ROS Production Using Fluorescent Microscopy

The production of ROS was determined using 2ʹ7ʹ-dichlorofluorescein diacetate (DCFH-DA; Life Technologies), a non-fluorescent and membrane-permeable probe. After oxidation, the probe converts into the highly fluorescent DCF. Hemocytes (1 × 10^5^ cells) cultured in 96-well plates were incubated with 10 µM DCFH-DA. ROS production was stimulated with 10 µg·mL^−1^ phorbol myristate acetate (PMA; Sigma-Aldrich, Saint Louis, MO, USA) [57]. Measurements of three replicates were conducted every 30 min for 90 min at 20 °C using a microplate reader (Sinergy H1, BioTek).

### 4.6. Transcription Expression of Candidate Genes

For qPCR analyses, 8 × 10^6^ hemocytes were incubated in 6-well plates with 100 nM STX for 4 or 16 h. Total RNA was extracted from each well using the TRIzol reagent (Life Technologies) following the manufacturer’s instructions. The quantity, purity and quality of isolated RNA were measured using a microplate reader (Synergy H1, BioTek). Subsequently, double-stranded cDNA was synthesized using the RevertAid H Minus First Strand cDNA Synthesis Kit (Thermo Scientific, Waltham, MA, USA). Moreover, five serial dilutions of cDNA stocks were conducted to establish the dynamic range for the genes under study. Enzyme efficiency between 90% and 110% in all reactions was obtained. Subsequently, a pool of samples from each experiment was used to select the best housekeeping gene according to its stability value inferred by the GeNorm application [58]. Three housekeeping genes were analyzed: *28S*, *elongation factor α* and *α-tubulin*, of which the latter was the most stable at the moment of analyzing its stability value. Subsequently, a gene expression analysis was conducted using the comparative ΔΔ*C*_t_ method, with 3 replicates for each experiment, and normalizing the expression data of each target gene with the *α-tubulin* endogenous control data. All reactions for dynamic range, endogenous validation and expression analysis were conducted using the Maxima Kit^®^ SYBR Green/ROX qPCR Master Mix (2×) (Fermentas, Waltham, MA, USA), according to the manufacturer’s instructions and using a primer concentration of 500 nM and a 1:5 dilution for each cDNA stock. qPCR was performed on an Eco Real-Time PCR System (Illumina, San Diego, CA, USA), and all gene expression analyses were performed using 3 biological replicates from each experiment. Specific primers were designed to amplify candidate genes related to immune response, such as antioxidant enzymes, oxidative phosphorylation transcripts, ion channels and pattern recognition receptors. Herein, *CAT*, *SOD*, *ferritin*, *metallothionein*, *NADH dehydrogenase 1* (*ND1*), *ATP6*, *cytochrome oxidase subunit I* (*COI*), *cytochrome oxidase subunit III* (*COIII*), *cytochrome B* (*CytB*), *voltage-dependent anion channel* (*VDAC*), *Ca^2+^ channel*, *K^+^ channel*, *C-type* *lectin* and *toll-like receptor* (*TLR*). The sequence information to design PCR primers and to evaluate their transcription expression was obtained from an EST-database generated by high-throughput sequencing from *M. chilensis* [59]. The list of oligonucleotide primers used to amplify target genes is shown in Table 1. Statistical analysis was performed using the Statistica software (Version 7.0, StatSoft, Inc., Tulsa, OK, USA).

**Table 1:** Gene names, forward and reverse primer sequences for real-time quantitative PCR (qPCR) analysis and references.

Gene	Primers (5ʹ–3ʹ)	Reference
*Catalase*	CCAGGTGTCCTTCCTGTTTTCT	Núñez-Acuña *et al.*, 2012 [60]
	TGTCCATCCTTGTTGACTGTCTTAA	
*Ec-SOD Cu/Zn*	TCGCTTTCAGTCAACAGAATGG	Núñez-Acuña *et al.*, 2012 [60]
	CCAAACTCGTGAACGTGGAA	
*Ferritin*	CAAGTGAACGCCATCAAGGA	Núñez-Acuña *et al.*, 2012 [60]
	CGTTGATGCTCTCTTTGTCATACA	
*Metallothionein*	CTGTGGTGACGCCTGCAAGT	Núñez-Acuña *et al.*, 2013 [20]
	CTGTGGTGACGCCTGCAAGT	
*NADH dehydrogenase I*	CTTACTAGGTGCAGTCCGTG	Primers designed in this study
	ACATAAACACGCCTGAAGATAGT	
*ATP6*	TGGGCATACCGCTGTGACTTACT	Primers designed in this study
	GTGCAGGGGCACCCATAGGA	
*Cytochrome oxidase subunit I*	TCAGCTGCTGTAGAGAGTGGGG	Primers designed in this study
	TCACGCCGGTGGTTCAGTTG	
*Cytochrome oxidase subunit III*	TATGTACCAGGCCCAAGTCC	Primers designed in this study
	AAAATCTGGGGGTTCGATGC	
*Cytochrome B*	GGAAGATGCCCGTTGGAGGC	Primers designed in this study
	CCCTGCGCTCCAGAAATTATGGG	
*Voltage-dependent anion channel*	CAGACAGGAAAGAAGCAGGG	Primers designed in this study
	CTAAGGCACCATTCACAGCT	
*Calcium channel*	TGTAAGCAAGTCGTGTGGGGCAC	Primers designed in this study
	ACGAGTTTGGGCAGACTATGATCCTG	
*Potassium channel*	CGGCAATTTGGACCGGGCTTGTG	Primers designed in this study
	GCGCCCTCTGTCCATTACTCGGC	
*C-type lectin*	GATATCGCTGTGCAGAACCA	Primers designed in this study
	AAACACAAGGCCAGTGGAAA	
*TLR*	CTTTCTTAGCCACGCCAGAC	Primers designed in this study
	TGTCATTGTCATTGTCAGTTTCGTCA	
*α-Tubulin*	GAGCCGTCTGCATGTTGAGC	Núñez-Acuña *et al.*, 2012 [60]
	TGGACGAAAGCACGTTTGGC	

### 4.7. Statistical Analyses

All statistical analyses were performed using the Statistica 10.0 (StatSfot) software. In order to assess the normality of the data, a Shapiro–Wilk test was conducted. When data followed a normal distribution, a one-way analysis of variance (ANOVA) followed by a *post hoc* Tukey HSD was performed. For non-parametric data, Kruskal–Wallis was used, followed by a Mann–Whitney test to compare between groups. Differences were considered significant when *p* < 0.05, represented with a, b, c, d superscripts or with an asterisk. In the case of relative gene expression, significant differences were represented with an asterisk.

## 5. Conclusions

In this study, we demonstrated the usefulness of a metabolically- and functionally-active primary culture of hemocytes in analyzing the effects of STX on the cellular and transcriptional response. Using primary cultures of hemocytes would reduce the interferences associated with handling fresh hemolymph. Cultured hemocytes were sensitive to STX, similar to fresh hemocytes analyzed *in vitro* and *in vivo*. Granulocyte-like cells were those principally affected by STX, suggesting that the toxin might be capable of modulating the cellular immune response in a way similar to other inductors, including foreign particles, pathogens and toxic microalgae. The transcriptional analysis used in this *in vitro* model could provide new perspectives for shellfish monitoring programs. However, more investigations are required.
